# *Cambridge Psycholinguistic Inventory of Christian Beliefs*: A registered report of construct validity, internal consistency and test–retest reliability

**DOI:** 10.3758/s13428-021-01632-3

**Published:** 2021-07-09

**Authors:** Kaili Clackson, Nadya Pohran, Riccardo M. Galli, Laura Labno, Miguel Farias, Tristan A. Bekinschtein, Valdas Noreika

**Affiliations:** 1 Consciousness and Cognition Lab, Department of Psychology, University of Cambridge, Downing Street, Cambridge, CB2 3EB UK; 2 Faculty of Divinity, University of Cambridge, Cambridge, UK; 3 Department of Experimental and Applied Psychology, Vrije Universiteit Amsterdam, Amsterdam, Netherlands; 4 School of Psychology, Bangor University, Bangor, UK; 5 Brain, Belief, and Behaviour Lab, Centre for Trust, Peace and Social Relations, Coventry University, Coventry, UK; 6 Department of Biological and Experimental Psychology, School of Biological and Chemical Sciences, Queen Mary University of London, London, UK

**Keywords:** Atheist, Belief, Christian, Moral, N400, Psycholinguistic inventory, Reaction times, Religious cognition, Scientific knowledge

## Abstract

While religious beliefs are typically studied using questionnaires, there are no standardized tools available for cognitive psychology and neuroscience studies of religious cognition. Here we present the first such tool—the *Cambridge Psycholinguistic Inventory of Christian Beliefs* (CPICB)*—*which consists of audio-recorded items of religious beliefs as well as items of three control conditions: moral beliefs, abstract scientific knowledge and empirical everyday life knowledge. The CPICB is designed in such a way that the ultimate meaning of each sentence is revealed only by its final critical word, which enables the precise measurement of reaction times and/or latencies of neurophysiological responses. Each statement comes in a pair of Agree/Disagree versions of critical words, which allows for experimental contrasting between belief and disbelief conditions. Psycholinguistic and psychoacoustic matching between Agree/Disagree versions of sentences, as well as across different categories of the CPICB items (Religious, Moral, Scientific, Everyday), enables rigorous control of low-level psycholinguistic and psychoacoustic features while testing higher-level beliefs. In the exploratory Study 1 (*N* = 20), we developed and tested a preliminary version of the CPICB that had 480 items. After selecting 400 items that yielded the most consistent responses, we carried out a confirmatory test–retest Study 2 (*N* = 40). Preregistered data analyses confirmed excellent construct validity, internal consistency and test–retest reliability of the CPICB religious belief statements. We conclude that the CPICB is suitable for studying Christian beliefs in an experimental setting involving behavioural and neuroimaging paradigms, and provide Open Access to the inventory items, fostering further development of the experimental research of religiosity.

**Supplementary Information:**

The online version contains supplementary material available at 10.3758/s13428-021-01632-3.

## Introduction

In psychological studies of religion, religious beliefs and attitudes are typically assessed by standardized questionnaires and scales, such as the Christian Orthodoxy Scale^1^ (Fullerton & Hunsberger, 1982) and the Francis Scale of Attitude toward Christianity (Francis & Stubbs, 1987). These and similar scales are successfully applied to study social and developmental areas of the psychology of religion, e.g. addressing topics such as religiosity and life satisfaction in older individuals (Hunsberger, 1985), attributional styles of religious individuals (Lupfer et al., 1988), parent-child dynamics in emerging religiosity (Leonard et al., 2013) or the association between religiosity and conservatism (Lewis & Maltby, 2000). However, despite a large number of questionnaires and scales available to study religiosity (for reviews, see Hill & Hood, 1999; Hill, 2013), there are no standardized tools available for behavioural and neuroimaging studies of religious cognition.

Neurocognitive studies typically rely on the accurate measurement of time between the presentation of independent factors, e.g. visual stimuli or auditory tones, and dependent factors, e.g. subsequent behavioural and neurophysiological responses, such as reaction times (RT) or latencies of brain responses such as event related potentials (ERP). In the context of cognitive studies of religion, verbal stimuli coinciding with an individual’s belief system are often expected to be more immediately accessible for cognitive evaluation, yielding faster RT. In this context, RT seems to reflect both the semantic complexity of the religious stimulus and the cognitive processes underlying religious beliefs, and has already been successfully employed in behavioural studies of religious cognition (Cavrak & Kleider-Offutt, 2015; Sharp et al., 2017; Wenger, 2004). However, single words are usually presented in such studies, such as God attributes (Sharp et al., 2017). While such studies are useful in assessing basic doctrinal beliefs, longer phrases and full sentences would be needed to express more complex forms of religious beliefs, such as “the Bible is the most important source of life instructions”. Unfortunately, there is no validated set of religious belief statements available for such studies.

Going beyond cognitive assessment-based research on religion, an increasing number of studies tap into the neural processes underlying religiosity (Brown & Strawn, 2012; McNamara, 2009; van Elk & Aleman, 2017). While most of the neural studies of religion focus on religious experiences and practices, such as mystical experiences and prayer in adult individuals (Beauregard & Paquette, 2006, 2008; Cristofori et al., 2016; Doufesh et al., 2012; Greyson et al., 2015), there is also a growing interest in the neural basis of religious cognition. For instance, several studies have investigated the N400 component, a negative ERP deflection peaking around 400 ms post stimulus in the central parietal area, in response to statements that contradict individual beliefs (Izzidien & Chennu, 2018; van Berkum et al., 2009). N400 is typically evoked by a semantically incongruous word that triggers a relatively low-level violation of the semantic prediction, e.g. “He took a sip from the transmitter” as opposed to “He took a sip from the waterfall” (Kutas & Hillyard, 1980). Importantly, a growing body of evidence suggests that N400 may also be triggered by a high-level linguistic violation of one’s world knowledge or individual belief system (Coronel & Federmeier, 2016; Fondevila et al., 2012, 2016; Hagoort et al., 2004; Lindeman et al., 2008; van Berkum et al., 2009). For instance, van Berkum et al. (2009) presented moral belief statements, such as “I think euthanasia is an acceptable/unacceptable course of action”, to members of a relatively strict Christian political party, and non-religious individuals. As predicted, value-inconsistent words elicited a negative N400 response, confirming that semantically congruous words that contradict individual moral beliefs modulate N400 amplitude.

Several studies have investigated whether paranormal and religious beliefs also modulate N400 responses (Fondevila et al., 2016; Izzidien & Chennu, 2018; Lindeman et al., 2008). For instance, Fondevila et al. (2016) reported N400 amplitude differences in response to intuitive sentences (e.g. “From his mind emerged the idea”), non-religious counter-intuitive sentences (e.g. “From his mind emerged the house”), and religious counter-intuitive sentences (“From his mind emerged the moon”), which were evaluated either literally or metaphorically. However, it is doubtful if participants perceived the last category of sentences as religious, as all of them were Spanish students, whereas sentences were extracted from non-Christian mythologies. Thus, rather than directly tapping into religious cognition, the study seemed to assess N400 dependency on different degrees of metaphors; for instance, ”religious'” counterintuitive sentences were rated more metaphorically than non-religious counterintuitive ones. Izzidien and Chennu (2018) used the N400 paradigm to assess the implicit responses of Muslim graduates to the test sentence “I believe Islamic law is fair”, which was presented multiple times within a longer list of control sentences. While all participants (*N* = 10) explicitly agreed with the test sentence, its N400 response resembled the N400 response to control sentences to which participants disagreed, pointing to an inconsistency between the explicitly held religious belief and its implicit neural processing. Unfortunately, the study is limited to the analysis of just one specific Muslim belief, whereas most psychology of religion studies focus on a broad range of Christian beliefs, experiences and practices (Hood et al., 2018).

 Currently, there is no validated inventory for carrying out behavioural and/or neuroimaging experiments of Christian beliefs. Such an inventory would allow researchers to measure response times and neurophysiological responses to belief statements and would enable a more mechanistic approach to religious cognition, thereby facilitating interaction between the cognitive science of religion and other fields of cognitive psychology and neuroscience. We propose that such an inventory should meet the following criteria:

1. *Belief statements should be audio-presented.* This avoids issues with individual differences in reading speed/skill (Bell & Perfetti, 1994), and allows for analyses to be time-locked to the presentation of specific words. Visual presentation techniques that would allow for similar timing accuracy (such as the moving-window paradigm) compromise on the naturalness of language processing. Thus, all sentences should be pre-recorded and audio-presented.

2) *The critical word should be placed at the end of the belief statement*. Response times indicating sentence comprehension and/or sentence plausibility judgment are commonly locked to the offset of the sentence, using both behavioural (Caplan & Waters, 2005) and electroencephalography (EEG) (Connolly et al., 1995) paradigms. The sentences should disambiguate at the end of the phrase, so that the meaning of the statement only becomes clear at the final word (Bekinschtein et al., 2011; Izzidien & Chennu, 2018).
3) *Each belief statement should have two orthogonal versions constructed with identical strings of words except for different critical words which lead to either Agree or Disagree responses.* Having identical strings of preceding words allows for the main body of the statement to be psycholinguistically and psychoacoustically matched across versions, thus ensuring that any behavioural and/or neural differences in response to Agree and Disagree sentences are due to the processing of the final critical words rather than content or auditory differences in the preceding parts of the sentence.
4) *Linguistic properties of critical words should be matched across Agree and Disagree statements.* To ensure that participants’ responses to Agree and Disagree versions of each item are not affected by the linguistic properties of the critical word, the Agree and Disagree versions of critical words should be matched for word frequency, length and sound intensity. To achieve this, each pair of beliefs statements should have a “sister pair” of statements with reversed association between critical words and Agree/Disagree responses, such that any response bias engendered by a particular critical word occurs equally in Agree and Disagree statements.

5) *There should be control conditions for religious belief statements.* RT or neural response differences between Agree and Disagree responses to belief statements, within and between Christian and Atheist participants, could be due to individual differences in language comprehension (Kidd, Donnelly, & Christiansen, 2018), or intelligence (Deary et al., 2001; Shcherbakova et al., 2017). Hence, in order to draw inferences specific to religious cognition, a set of statements in another domain of abstract thought would be needed as a control condition, such as moral beliefs, abstract scientific knowledge or empirical facts about everyday life.
6) *Properties of critical words should be matched across categories.* To allow for a fair comparison of responses to statements in experimental and control conditions, the critical words should be matched across conditions for word frequency, length and sound intensity.

Aiming to facilitate behavioural and neuroimaging research of religious cognition, we have developed a new inventory of Christian beliefs, the *Cambridge Psycholinguistic Inventory of Christian Beliefs* (*CPICB*), which meets the aforementioned requirements. We have piloted it behaviourally with 20 participants (10 Christians and 10 Atheists—see Methods for definitions of participant groups) and found high internal consistency and construct validity. In the following, we (i) describe the development of the CPICB, (ii) report exploratory pilot results of its construct validity and internal consistency (Study 1), (iii) describe the experimental design of the confirmatory study with new groups of 20 Christians and 20 Atheists (Study 2), and (iv) report the confirmatory assessment of the construct validity, internal consistency and test–retest reliability of the CPICB (Study 2). The data analysis plan for Study 2 was registered at the OSF Registries before data analysis began, see https://osf.io/vrnxg/.

## Study 1: The development and exploratory assessment of the *Cambridge Psycholinguistic Inventory of Christian Beliefs*

In Study 1, we created and tested an extended version of the inventory (480 items), aiming to remove problematic statements before carrying out a confirmatory Study 2 with a shorter final version of the inventory (400 items).

## Methods

### Inventory statements

The first version of the inventory consisted of 480 statements, of which 120 related to Christian belief (or lack thereof; to be returned to in more detail below), 120 to moral beliefs (these are generally agreed-upon aspects of good/bad behaviour rather than debated topics such as euthanasia or abortion), 120 to abstract scientific knowledge (specifically scientific statements that are accepted as fact but which a non-specialist individual cannot empirically test, instead relying on expert opinion), and 120 to everyday knowledge (i.e. information that most people acquire through direct experience). These categories were selected to reflect beliefs and knowledge on a scale from individual (personal beliefs) to factual (accepted by all). Religious beliefs are very personal and vary from individual to individual even within a seemingly-singular faith community, moral beliefs are generally agreed upon at a societal level, abstract scientific knowledge is generally accepted as a “fact” but can change as new discoveries are made, and everyday knowledge relates to facts learned from personal experience. The statements were designed to elicit a response detailing the participant’s level of agreement with the statement on a 5-point scale (strongly disagree to strongly agree). Items in the Religious category were created such that each statement is expected to elicit opposite responses from orthodox Christians and Atheists, while items in the other categories are expected to elicit the same responses from both groups.

Statements were created in sets of four, consisting of two “sister” pairs. Within each pair, one version is expected to generate an “agree” response, and one a “disagree” response and between sister pairs the critical-word-to-expected-response pairing is reversed as shown in Tables S1 and 1.

Within each pair, the two sentences were identical except for the critical word, which determined the expected response. All four statements shared a “baseline” segment (the word or words before the critical word), and sister pairs featured the same critical words but framed so that each critical word generated the opposite expected response. This was done to avoid the possibility that responses would be affected by the features of the particular critical word (e.g. word familiarity) through ensuring that all features of critical words (frequency, length, familiarity, etc.) were matched between the Agree and Disagree items. Items in each condition covered a range of topics, as shown in Table S2.

Critical words were a maximum of three syllables long, and the mean length (in syllables) and frequency (as measured by the SUBTLEX frequency database based on subtitles of British television programs; Van Heuven et al., 2014) was approximately matched (pending full matching following selection of the final 400 items for Study 2) across conditions.

### Inventory development

During the development of the CPICB inventory, draft versions were sent to three groups of experts for feedback and comments.

- The Religious items list was first sent to lay members of different Christian denominations (Anglican, Pentecostal, Adventist), asking them to report whether they agreed with the individual items provided. Following this, the Religious items list was sent to both Christian and Atheist “experts” who provided feedback on the content of the statements, particularly whether they felt that most Christians/Atheists they encountered day to day would be likely to give a response in the expected direction. These experts included local priests, ministers and priests-in-training from a variety of Christian denominations, including the Anglican, Roman Catholic and Baptist churches in Cambridge, as well as leaders of both national and local atheist/sceptic organisations, such as Humanists UK.
- The Scientific items list was sent to University of Cambridge scientists working in each of the fields covered (astronomy, physics, Earth science, biology, medicine), who were asked to check the factual content of statements and give their comments.
- The full list of items (from all conditions) was sent to four native English speakers (a mixture of Christians and Atheists) who provided feedback on the general intelligibility of the items.

Several rounds of revisions were made to the items following feedback from these three groups.

### Recording of sound files

Items were recorded for auditory presentation by a female native English speaker using specialist recording equipment in a soundproof booth. Following recording, sound files were spliced (using Audacity software) so that the main part of the sentence was identical for each pair and the baseline segment (minimum length 110 ms) was identical across all four versions, providing a sufficiently long time window for the 100 ms ERP baseline. The two instances of each critical word were also identical. This ensured that items in any pair differed only in the critical word, and that across sister pairs, the baseline + critical word was identical across Agree and Disagree versions, as shown in Table 1.

**Table 1:**
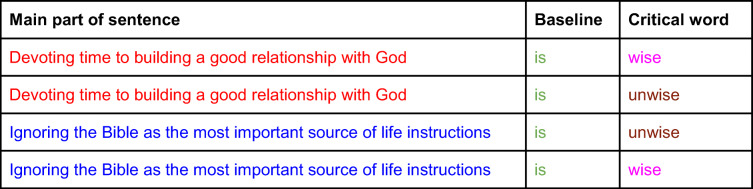
Sentence pairing and splicing of audio files

**Note:** Colours show how identical audio files were spliced together so that each sentence pair (red and blue) was identical up to the point of the critical word, and the two sister pairs were identical from the start of the baseline (green) to the end of critical words (magenta and brown)

In some item pairs the length of the baseline as recorded was naturally longer in one version than another due to the differing onsets of critical words. In such cases, the duration of the baseline was manipulated so as to be somewhere between the two natural lengths, and sound files were carefully spliced so as to maintain the natural rhythm and sound of the sentence while maintaining the splicing formula shown in Table 1. In order to avoid an occasional acoustic noise at the onset or offset of sentences, we inserted a 100 ms silent gap at the beginning of each item, and a 15 ms silent gap at the end of each item. Spliced sound files were then equalised to a mean intensity of 70 dB using PRAAT software.

### Participants

The main aim of Study 1 was to identify which inventory items successfully elicited the responses we expected from the two groups, in order that we could then select the most successful items for the final list used in Study 2. For this, we specifically recruited participants with strong Christian/Atheist views by advertising our study on the online portal of Cambridge Psychology Research and university advertisement boards, local churches and atheist societies, as well as by sending an invitation to an email list of the Faculty of Divinity at the University of Cambridge and through word of mouth. All participants were aged 18–45 and identified themselves as native speakers of English. Volunteers completed an online screening questionnaire, and 20 participants—10 Christians and 10 Atheists—were selected from amongst those who met four criteria for being either a Christian or an Atheist: (1) self-identification, (2) responses on the Christian Orthodoxy Scale (COS) (Fullerton & Hunsberger, 1982), (3) responses to a question about belief in God from the International Social Survey Programme^2^, and (4) frequency of taking part in religious practices, see Table S3.

The selected 20 participants (14 female; age range 18 to 45 years, *M* = 25.3, *SD* = 6.8) were matched by age (Christians: *M* = 25.1 years; Atheists: *M* = 25.6 years; independent-samples *t* test: *t*(2,18) = 0.16, *p* = 0.88) and strength of orthodox Christian beliefs or their rejection (Christians: *M* = 62.1, Atheists: *M* = -62.4; independent-samples *t* test of absolute values: *t*(2,18) = 0.09, *p* = 0.93). We asked Christian participants to self-identify which denomination (or none) they belonged to, and we received a mix of responses indicating that our participants were spread out across "Anglican" (3), "Evangelical" (1), "Protestant" (3), "Orthodox" (1), and "Catholic" (2).

Participants signed an informed consent form and took part in a behavioural pilot experiment, for which they were compensated at a rate of £10 per hour. The study protocol was approved by the Cambridge Psychology Research Ethics Committee (approval no.: PRE.2018.040). The study was carried out in accordance with the guidelines of the Helsinki declaration for the treatment of human participants.

### Behavioural testing

The experiment took place in one of the behavioural testing rooms of the Department of Psychology at the University of Cambridge. The room was equipped with a laptop (Apple Inc.) connected to a 22-inch LCD screen, a standard QWERTY keyboard, a pair of headphones, a desk and a chair. The experiment was programmed using MATLAB (MathWorks, Inc.) with the Psychophysics Toolbox version 3 (PTB-3) (Brainard, 1997; Kleiner et al., 2007; Pelli, 1997).

**Fig. 1:**
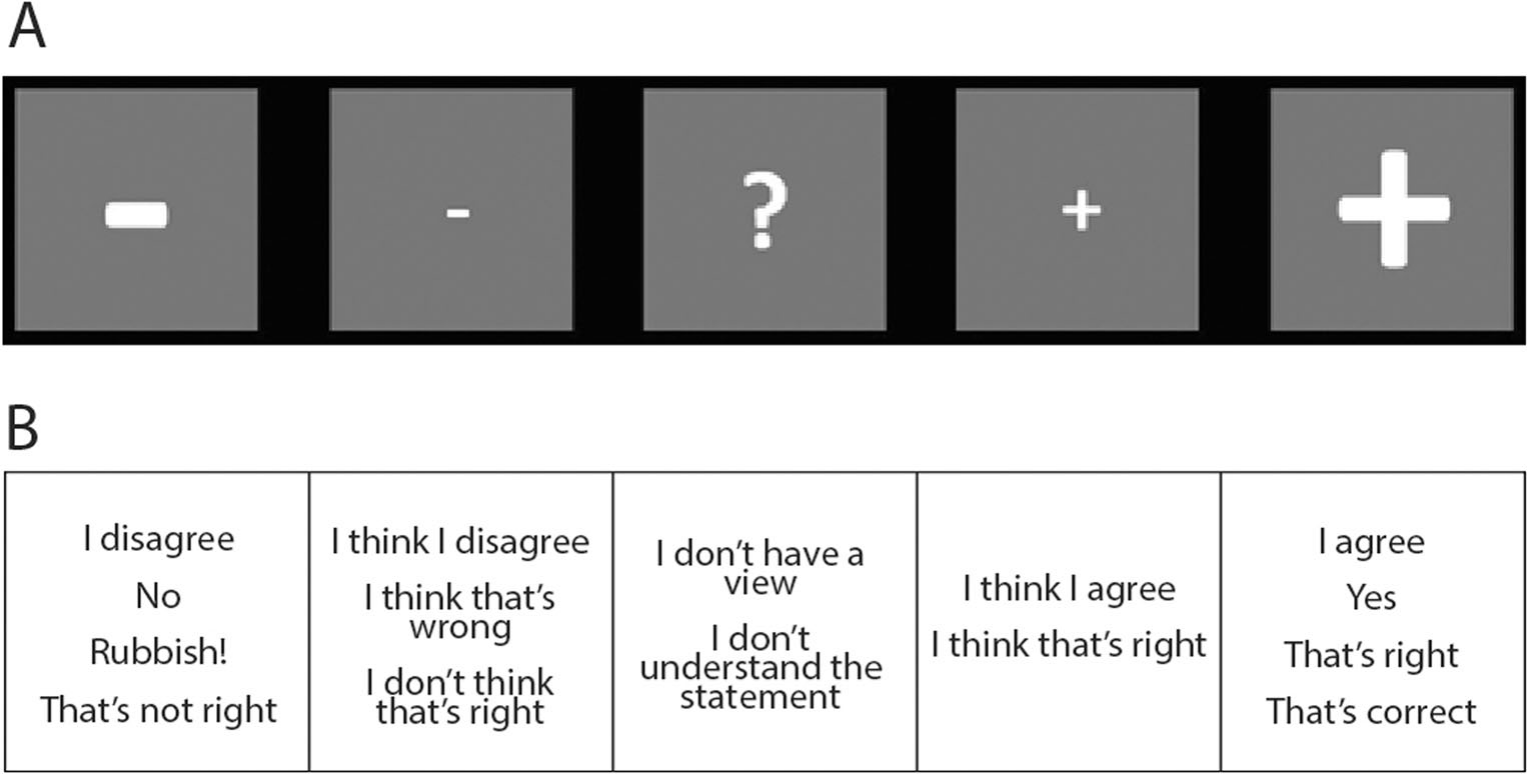
Visual depiction and instructed meaning of response keys. **a** Image of the answer keys that appeared on screen to prompt participants to provide a response. **b** Provided interpretation for each of the possible answer keys

At the beginning of the session, participants completed the Edinburgh Handedness Inventory (Oldfield, 1971) and were talked through the possible responses to the sentences they would hear in the main task, from strong disagreement to strong agreement (see Fig. 1). Importantly, the balanced numbers of Christian and Atheist versions of the Religious statements, as well as an equal proportion of Agree and Disagree statements in the Moral, Scientific, and Everyday categories enable control of a very liberal or conservative response style, i.e. consistently weak or strong agreement or disagreement with statements.

A variety of interpretations were given for each response key, as some statements required a response that related to personal opinion whereas some related to factual correctness. Participants were specifically asked to respond according to their views in their “heart of hearts”, not limiting themselves to expressing views that they felt were publicly acceptable, or views endorsed by their church, society or family. We emphasised that we were not looking for what might be seen as the “majority view” of any community that they identified with; we instead wanted to know their unique opinion. For example, it was emphasised that agreeing with the statement “People who believe in X are wrong” is not the same as saying that people should not believe in X, or that one would tell a believer of X that they are wrong.

During testing, participants sat at a distance of 70 cm from the computer screen. A single trial consisted of the presentation of a grey fixation dot on the screen and concomitant delivery of the audio statement through headphones, followed by the presentation of an image representing the five possible response keys (as shown in Fig. 1a), which prompted the participant to give a response. Participants were instructed to look at the grey dot on the screen while listening to the statement, and to keep their dominant index finger at a fixed location just below the keyboard. When the response key image appeared on the screen, they should move their finger and press one of the response keys. The keyboard was covered so that only the relevant five response keys were visible. Participants were instructed to answer as quickly as possible, basing their responses on gut feeling rather than long consideration. The next trial was initiated immediately after the participant’s response. If no response was given after 5 seconds, the next trial commenced.

Before commencing the main task, participants completed a practice block of 16 items to become familiar with the protocol. These practice items were designed to reflect the diversity of topics and linguistic structures found in the experimental items. The main task was delivered in 10 blocks of 48 items, which were presented in a randomized order, and participants were encouraged to take breaks between blocks.

## Results

All 20 participants completed the task.

### Item pruning

To identify the best 25 sets of items (out of 30) in each category (religious, moral, scientific, everyday) to be included in the final set, we calculated for each item how many of the 20 pilot participants had given the “expected” response for their group (either strongly or weakly). Item sets were then ranked by the lowest “expected response” score of any individual item in that set, and lowest-scoring sets were discarded. Where there were a number of sets with the same lowest score, between-condition matching on critical word frequency and critical word length in syllables was taken into account. Of the items selected to remain in the inventory, the lowest number of expected responses to any item (out of 20) was 13 for Religious items (*M* = 18.24, *SD* = 1.66), 17 for Moral items (*M* = 19.36, *SD* = 0.81), 14 for Scientific items (*M* = 18.58, *SD* = 1.57), and 18 for Everyday items (*M* = 19.59, *SD* = 0.68).

### Item matching

Following selection of the final list of 400 items, independent *t* tests confirmed that there were no significant differences between critical words across the four categories with reference to word frequency, intensity (dB), and length in syllables (see Table 2).

**Table 2:** Results of *t* tests comparing linguistic and acoustic properties of critical words and sentences between categories

	Religious vs Moral	Religious vs Scientific	Religious vs Everyday	Moral vs Scientific	Moral vs Everyday	Scientific vs Everyday
**Critical word frequency**	*t*(198) = −.22 *p* = .83	*t*(166.21) = .70 *p* = .48	*t*(164.84) = .02 *p* = .99	*t*(168.56) = .98 *p* = .33	*t*(167.18) = .28 *p* = .78	*t*(198) = −.92 *p* = .36
**Critical word intensity (dB)**	*t*(198) = 1.31 *p* = .19	*t*(198) = −0.10 *p* = .92	*t*(198) = 1.34 *p* = .18	*t*(198) = −1.28 *p* = .20	*t*(198) = .09 *p* = .93	*t*(198) = 1.31 *p* = .19
**Critical word length (syllables)**	*t*(198) = 1.25 *p* = .21	*t*(198) = .73 *p* = .47	*t*(191.87) = .38 *p* = .71	*t*(198) = −.56 *p* = .57	*t*(195.16) = −.97 *p* = .33	*t*(198) = −.40 *p* = .69
**Critical word length (ms)**	*t*(198) = 0.61 *p* = 0.55	*t*(198) = 1.29 *p* = .20	*t*(198) = 2.72 ***p*** **= 0.007**	*t*(198) = .63 *p* = .53	*t*(198) = 1.97 ***p*** **= 0.05**	*t*(198) = 1.38 *p* = 0.17
**Length of sentences (words)**	*t*(198) = −.43 *p* = .67	*t*(198) = −.91 *p* = .36	*t*(198) = 1.91 ***p*** **= .057**	*t*(198) = −.49 *p* = .63	*t*(198) = 2.32 ***p*** **= .021**	*t*(198) = 2.76 ***p*** **= .006**

However, we noted that items of Everyday knowledge had a significantly shorter length of critical words in milliseconds and a shorter length of sentences in words than items of the other three categories (see Table 2). In case this might be of concern for some studies, we ascertained that it is possible to replace 20 items from the Everyday category of the main set of 400 items (4031:4034, 4141:4144, 4191:4194, 4251:4254, and 4291:4294) with 20 items from the Everyday category of the excluded set of 80 items (4111:4114, 4151:4154, 4171:4174, 4261:4264, and 4271:4274). This yields no significant differences in the linguistic and acoustic properties of critical words and sentences between conditions (see Table S4). Thus, if needed, these or other extra items could be used when matching various psycholinguistic properties across categories and conditions of interest, depending on the needs of the particular research focus. In Study 2, we used the original set of 400 items that were selected based on the number of expected responses.

In order to ensure that the splicing process had not yielded sentences with acoustically unnatural properties which could affect participants' behavioural and electrophysiological responses, four linguistically naive native English speakers listened to the 400 selected statements in randomised order and indicated if they detected anything noticeable about the recording. 387/400 items were rated by all raters as sounding completely natural. For 12 items (spread across all four conditions) a splice junction was identified by one rater, and for one item a splice junction was detected by two raters. As these were responses from raters who had been specifically instructed to listen for anomalies in the recordings, we were satisfied that the splicing effects would be negligible when testing participants who were focused on the meaning, rather than the sound quality of the sentences. Following each pilot testing session, participants were prompted to give their thoughts on the task they had just completed, and none mentioned noticing anything about the acoustic quality of the spoken stimuli.

### Construct validity of the Cambridge Psycholinguistic Inventory of Christian Beliefs

To assess the construct validity of the averaged Religiosity score of the CPICB, the scores were correlated with the summed scores of the Christian Orthodoxy Scale (Fullerton & Hunsberger, 1982), which is a widely used scale with strong psychometric properties that assesses orthodoxy of Christian beliefs (Adams et al., 2018; Altemeyer & Hunsberger, 1992; Johnson et al., 1993; Lupfer et al., 1988; Pancer et al., 1995; Sanders et al., 2015; Truelove & Joireman, 2009). In the standard coding, the Christian Orthodoxy Scale summed scores range from 24 to 168, with 96 marking the centre point that separates Christian and Atheist responses. For ease of use we have rescaled this score so that it ranges from −72 to +72, with 0 as the centre point, a minus score shows a generally Atheist outlook, and a plus score a Christian one, with larger absolute numbers denoting stronger views.

Individual responses to the selected 100 Religious items of the CPICB ranged between −2 (“I disagree”) and +2 (“I agree”). Given that half of the religious statements were designed to elicit Christian Agree/Atheist Disagree responses, while the other half of the religious statements were designed to elicit Christian Disagree/Atheist Agree responses, the average score would have been close to 0 for both groups of participants. We thus reversed the positive or negative sign of Christian Disagree/Atheist Agree statements for all participants, after which a mean score close to +2 indicated that the participant usually responded with a strongly Christian response, i.e. selecting “I agree” for Christian Agree items or “I disagree” for Christian Disagree items. Again, a mean score close to −2 showed more Atheist responses, i.e. selecting “I agree” for Atheist Agree items or “I disagree” for Atheist Disagree items. The absolute values of mean scores denoted the strength of views.

**Fig. 2:**
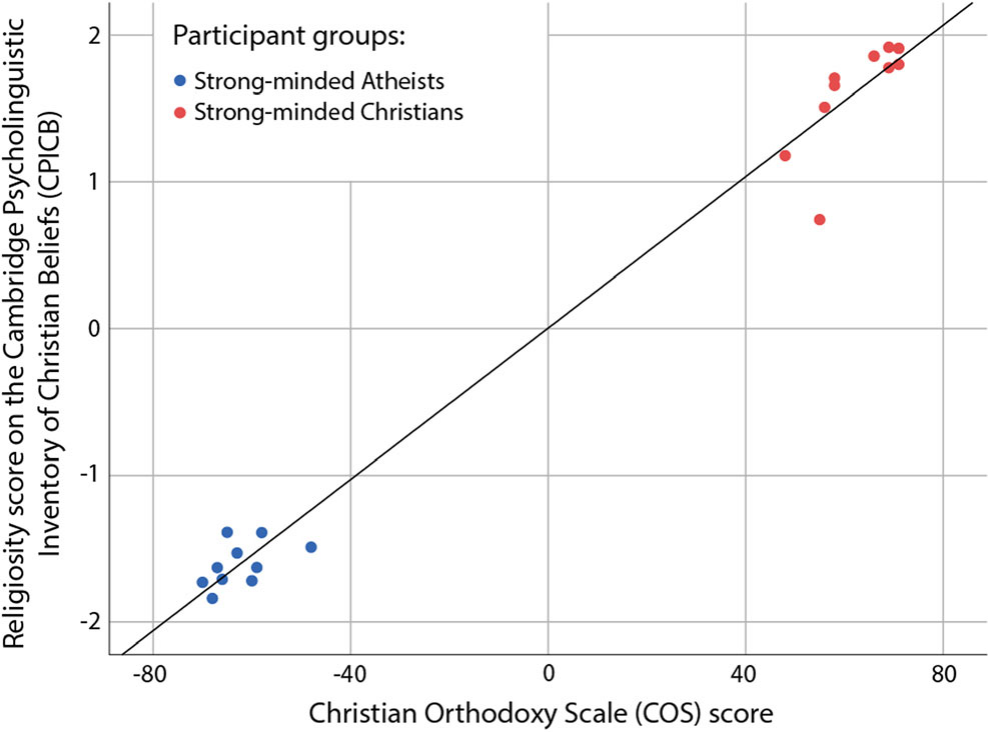
Association between religiosity scores of the Christian Orthodoxy Scale and the Cambridge Psycholinguistic Inventory of Christian Beliefs (*N* = 20, Study 1)

Given that our participants were specifically recruited as having extreme scores (< −45 or > +45) on the Christian Orthodoxy Scale, the data were not normally distributed, so the non-parametric correlation was calculated. We observed a significant positive association between the two measures of religiosity (Spearman’s rho = 0.942, *p* < 0.001, see Fig. 2), providing preliminary confirmation of the construct validity of the Religious beliefs category of the CPICB.

### Internal consistency of the Cambridge Psycholinguistic Inventory of Christian Beliefs

To assess the internal consistency of the CPICB, i.e. whether different sentences measure the same concept, we computed Cronbach’s alpha separately for the Religious, Moral, Scientific and Everyday categories of the inventory. Cronbach’s alpha indicates how closely related a set of items are as a group: < 0.70 is regarded as poor or unacceptable, 0.70–0.79 as fair, 0.80–0.89 as good, and ≥ 0.90 as excellent (Cicchetti, 1994).

Given that Cronbach’s alpha is sensitive to missing data, we replaced missing values for individual responses with the mean of the whole group for the Moral, Scientific and Everyday categories, whereas, when replacing missing values in the Religious section, means were calculated separately for Christians and Atheists. Across 400 inventory items and 20 participants (8000 data points), only 42 responses (0.53% of the data) were missing (see Table S5), confirming the feasibility of the materials and study design, specifically, that participants were able to process the sentences and respond within the allocated response period of 5 seconds.

Given that half of the sentences were designed to produce “I agree” and another half “I disagree” responses, inconsistent positive and negative correlations would have distorted the assessment of internal consistency. Thus, before calculating Cronbach’s alpha, the positive/negative sign of Disagree item responses was reversed.^3^ For the Religious category, Christian Disagree/Atheist Agree items were reversed. A significant proportion of items yielded identical responses from all participants, e.g. all of them disagreed with the item and chose a −2 response, and hence had zero variance. Such items were removed from the scale before calculating Cronbach’s alpha (see Table 3). For the remaining items, we observed excellent internal consistency (Cronbach’s alpha > 0.90) for both individual sections of the inventory as well as the full inventory when all participants were analysed together (see Table 3). Subgroup analysis indicated lower internal consistency for the Atheist group (Cronbach’s alpha = 0.76).

**Table 3:** Internal consistency of the Cambridge Psycholinguistic Inventory of Christian Beliefs (Study 1)

	**Religious**			**Moral**	**Scientific**	**Everyday**	**Full inventory**
	Christian	Atheist	All				
**Participants,** ***N***	10	10	20	20	20	20	20
**Items,** ***N***	100	100	100	100	100	100	400
**Items excluded,** ***N***	3	25	0	12	10	37	59
**Cronbach’s alpha**	0.96	0.755	0.998	0.954	0.94	0.934	0.987

## Study 2: The confirmatory assessment of the *Cambridge Psycholinguistic Inventory of Christian Beliefs*

### Aims of the study

Encouraged by the preliminary findings of Study 1, we aimed to replicate them with a larger sample of participants. Specifically, given that only relatively strong Christians and Atheists were tested in Study 1, we wanted to extend inventory assessment to more moderate Christians and Atheists. Furthermore, in addition to the assessment of construct validity and internal consistency of the inventory, we aimed to assess its test–retest reliability.

### Participants

Forty participants were selected from a larger database of > 350 participants willing to take part in studies on religiosity, aiming to represent the full continuum of beliefs from strongly Christian to strongly Atheist, i.e. including those with middle-ground beliefs, unlike in Study 1. As before, participants were recruited by advertising our study on the online portal of Cambridge Psychology Research and university advertisement boards, local churches and atheist societies, as well as by sending an invitation to an email list of the Faculty of Divinity at the University of Cambridge, and through word of mouth. Interested individuals completed the same online screening questionnaire as those taking part in Study 1. Two participants could not complete the second session of the study due to medical reasons and were replaced by another two volunteers.

Ten participants were recruited to each of four groups: Strong-Minded Atheists (Christian Orthodoxy Scale scores of −45 to −72), Moderate Atheists (COS scores of 0 to −45), Moderate Christians (COS scores of 0 to 45) and Strong-Minded Christians (COS scores of 45–72). Contrary to Study 1, participation in Christian practices and self-identification as Christian or Atheist was not taken into account as there was considerable variation among the “moderate” groups, with some participants’ positive or negative direction of COS scores not matching their self-identification, and some considering themselves agnostic or spiritual rather than Atheist or Christian. Among Strong-Minded Christians, participants self-identified as “Anglican” (5), “Newfrontiers” (2), “Evangelical Lutheran” (1), “Catholic” (1) and “Quaker/Anglican” (1). Among Moderate Christians, participants reported belonging to “Anglican” (3), “Protestant” (1), “Salvation Army” (1), “Orthodox” (1) and “Catholic” (2) denominations, whereas two participants did not report denominational identity. Among Moderate Atheists, one reported being “Anglican” and one being “Orthodox”.

A *t* test comparing the absolute COS scores of Atheists (46) and Christians (45.6) (pooling across Moderate and Strong-Minded groups in each case) showed no significant difference (*t*(38) = 0.57, *p* = 0.995). Likewise, there were no significant group differences in the absolute COS scores between Strong-Minded Atheists (64.4) and Strong-Minded Christians (65.3) (*t*(18) = −0.30, *p* = 0.77) or between Moderate Atheists (27.5) and Moderate Christians (25.8) (*t*(18) = 0.28, *p* = 0.78).

All participants were aged 18–45 and identified themselves as native speakers of English. A one-way ANOVA to test for group differences showed no significant differences in age (*F*(3,36) = 0.45, *p* = 0.72) or verbal IQ (*F*(3,36) = 0.37, *p* = 0.78). There were comparable gender ratios between participant groups, with 2–4 male participants in each group. Details of participants in the four groups are given in Table S6.

The study protocol was approved by the Cambridge Psychology Research Ethics Committee (Approval No: PRE.2018.040), and it was carried out in accordance with the guidelines of the Helsinki declaration for the treatment of human participants. Participants were compensated at a rate of £10 per hour.

### Inventory statements

Based on the findings of Study 1, the final version of the inventory used in Study 2 consisted of 400 statements of which 100 related to Christian beliefs (or lack thereof), 100 to moral beliefs, 100 to scientific abstract knowledge and 100 to everyday knowledge, which were selected from the first version of the inventory consisting of 480 statements (see Study 1). The remaining statements were kept in the original sets of four, consisting of two “sister” pairs (see Table 1). Critical words of statements were a maximum of three syllables long, and there were no significant linguistic and psychoacoustic differences between critical words across the four conditions (Religious, Moral, Scientific, Everyday) with reference to frequency, length in syllables and intensity (dB) (see Table 2).

### Behavioural testing

Individual participants took part in two sessions of behavioural testing, which were carried out by the same experimenter using the same equipment in the same lab. We planned the delay between visit 1 and visit 2 to last between two weeks and two months. Most of the participants preferred the shortest possible delay and there were on average 18.5 days (*SD* = 7.1) between visits 1 and 2. A one-way ANOVA to test for participant group differences showed no significant differences in test–retest interval between the two sessions (*F*(3,36) = 0.83, *p* = 0.49; see Table S6).

As for Study 1, the experiment took place in one of the behavioural testing rooms of the Department of Psychology at the University of Cambridge. The room was equipped with a laptop (Apple Inc.) connected to a 22-inch LCD screen, a standard QWERTY keyboard, a pair of headphones, a desk and a chair. The experiment was programmed using MATLAB (MathWorks, Inc.) with the Psychophysics Toolbox version 3 (PTB-3) (Brainard, 1997; Kleiner et al., 2007; Pelli, 1997).

At the beginning of the first session, participants signed informed consent forms and completed the Edinburgh Handedness Inventory (Oldfield, 1971), after which they read instructions about the study and were talked through the possible responses to the sentences they would hear in the main task (see Fig. 1). The experimental procedure was identical to Study 1 (see above), except that due to a smaller number of items the main task was delivered in 10 blocks of 40 items, which were presented in a randomized order.

During the second session, participants read the same instructions about the study and were talked through the possible responses to the sentences they would hear in the main task in the same way as they were introduced to the task during the first session. Afterwards, participants carried out the practise block with the same 16 sentences, followed up by the main task delivered in 10 blocks of 40 items, and they were again encouraged to take breaks between blocks. Items were presented in a randomized order, which was generated independently of the first session. Once participants completed the task, the experimenter asked them to fill in the Cognitive Reflection Test (Frederick, 2005)^4^ and administered verbal IQ sections (Vocabulary and Similarities) of the Wechsler Abbreviated Scale of Intelligence, Second Edition (WASI-II; Wechsler, 2011).

The data analysis plan for Study 2 was preregistered at the OSF Registries, as described in the following. For more details, see https://osf.io/vrnxg/

### Construct validity of the Cambridge Psycholinguistic Inventory of Christian Beliefs

To assess the construct validity of the averaged Religiosity score of the CPICB, it was correlated with the summed scores of the Christian Orthodoxy Scale (Fullerton & Hunsberger, 1982). Similarly to Study 1, the COS score was rescaled to range from −72 to +72, with 0 as the centre point, a minus score showing a generally Atheist outlook, and a plus score a Christian one, with larger absolute numbers denoting stronger views. For the Religious category of the CPICB, a mean score close to +2 indicates that the participant responded with a strongly Christian response, whereas a mean score close to −2 shows strongly Atheist responses.

Given that the 40 participants were specifically recruited to cover different ranges of the Christian Orthodoxy Scale, their Christian Orthodoxy Scale summed scores had a platykurtic distribution with flat tails (excess kurtosis = −1.52) and did not appear to be normally distributed (Shapiro-Wilk test: *W* = 0.90, *p* = 0.002). Thus, non-parametric Spearman’s rank-order correlation was calculated to assess the construct validity of the Religiosity score of the CPICB.

### Internal consistency of the Cambridge Psycholinguistic Inventory of Christian Beliefs

To assess the internal consistency of the CPICB, i.e. whether the 100 sentences within each category measure the same concept, we computed Cronbach’s alpha separately for the Religious, Moral, Scientific and Everyday sections of the Inventory and for the full inventory. Cronbach’s alpha < 0.70 was regarded as poor or unacceptable, 0.70–0.79 as fair, 0.80–0.89 as good and ≥ 0.90 as excellent (Cicchetti, 1994).

Given that Cronbach’s alpha is sensitive to missing data, we replaced missing values for individual responses with the mean of the whole group (*N* = 40) for the Moral, Scientific and Everyday sections. When replacing missing values in the Religious section, means were calculated separately for each subgroup of Christians and Atheists (*N* = 10).

Given that half of the sentences were designed to produce “I agree” and another half “I disagree” responses, inconsistent positive and negative correlations would have distorted the assessment of internal consistency. Thus, before calculating Cronbach’s alpha, the negative/positive sign of Disagree items was reversed for the Moral, Scientific and Everyday sections of the inventory. Likewise, the negative/positive sign of Christian Disagree/Atheist Agree items was reversed for the Religious section of the inventory. Items with identical responses from all participants were removed from the scale before calculating Cronbach’s alpha.

### Test–retest reliability of the Cambridge Psycholinguistic Inventory of Christian Beliefs

To assess test–retest reliability of the mean scores within each category of the inventory (Religious, Moral, Scientific and Everyday), a two-way mixed model intraclass correlation coefficient of an absolute agreement type (ICC 3.1) was used to compare performance on sessions 1 and 2 (Koo & Li, 2016; Shrout & Fleiss, 1979). Reliability with ICC values < 0.40 was regarded as poor, 0.40–0.59 as fair, 0.60–0.74 as good, and ≥ 0.75 as excellent (Cicchetti, 1994). In addition to the ICC, we report the 95% confidence interval, and F statistic with true value 0 and its significance (p).

For each session, a mean response score was calculated separately for the Religious, Moral, Scientific and Everyday sections of the inventory. Given that half of the statements were designed to elicit Agree responses, while the other half of the statements were designed to elicit Disagree responses, the average score would be close to 0 for all groups of participants. We thus have reversed the positive or negative sign of (Christian) Disagree statements for all participants. In the Religious category, a mean score close to +2 indicated that the participant usually responded with a strongly Christian response, whereas a mean score close to −2 showed more Atheist responses. In addition to the mean scores over both agreement conditions, we reported test–retest reliability statistics separately for the (Christian) Agree and (Christian) Disagree items.

## Results

### Construct validity of the Cambridge Psycholinguistic Inventory of Christian Beliefs

**Fig. 3:**
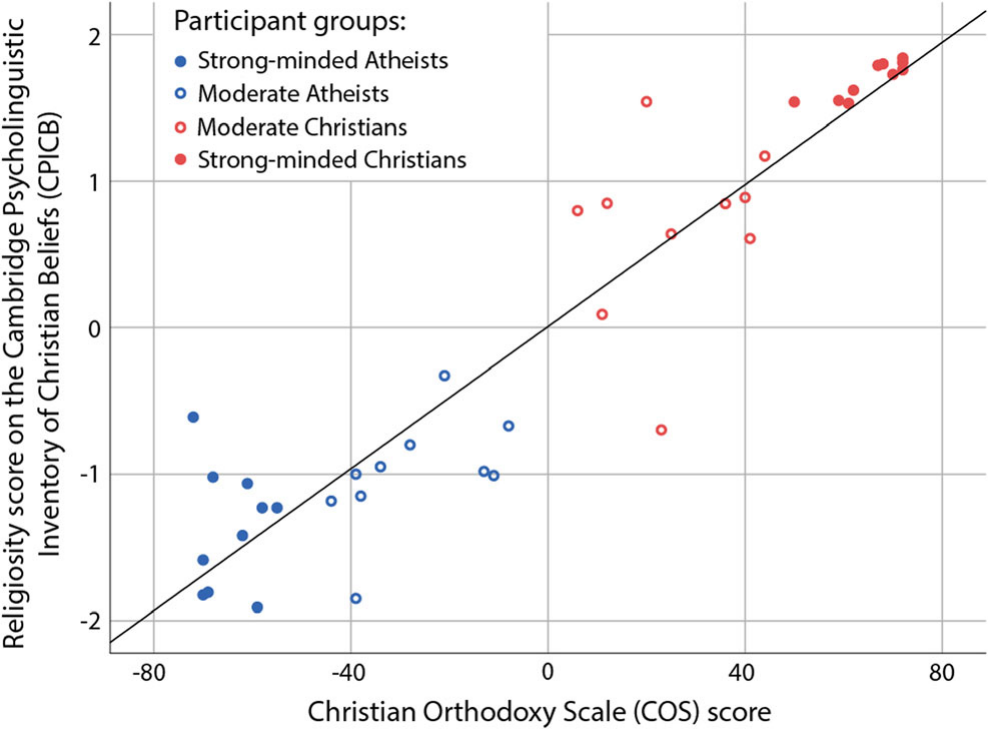
Association between religiosity scores of the Christian Orthodoxy Scale and the Cambridge Psycholinguistic Inventory of Christian Beliefs (*N* = 40, Study 2)

We observed a significant positive correlation between the COS and the CPICB measures of religiosity (Spearman’s rho = 0.915, *p* = 1.5E−16, see Fig. 3), confirming the construct validity of the Religious beliefs category of the CPICB.

### Internal consistency of the Cambridge Psycholinguistic Inventory of Christian Beliefs

Across 400 inventory items and 40 participants (16000 data points), only 82 responses (0.51% of data) were missing (see Table S7), confirming the feasibility of the materials and study design, specifically, that participants were able to process the sentences and respond within the allocated time. Regarding individual inventory items that participants did not respond to on time, most were missed by just one or two participants. In the Religious category, two items were missed by three participants (items 1123 and 1293) and one item was missed by four participants (item 1273). In the Scientific category, item 3013 was missed by three participants.

We observed excellent internal consistency (Cronbach’s alpha ≥ 0.90) at a full group level (*N* = 40) for both individual sections of the inventory as well as the full inventory (see Table 4). When the internal consistency of Religious items was tested separately for each subgroup, it was found to be excellent for Strong-Minded Atheists, Moderate Atheists and Moderate Christians (Cronbach’s alpha ≥ 0.94), but poor or unacceptable for Strong-Minded Christians (Cronbach’s alpha = 0.57). This was caused by a small number of items which elicited unexpected responses from this group. After excluding two of the problematic items (1162: “People who believe that the birth of Jesus was predicted in advance are wrong”, and 1173: “The claim that, after death, human existence ceases completely is unlikely”), fair internal consistency was observed for the remaining Religious items in the Strong-Minded Christian subgroup (Cronbach’s alpha = 0.71). Good internal consistency was observed for the remaining Religious items in the Strong-Minded Christian subgroup (Cronbach’s alpha = 0.80) when an additional four problematic items were excluded (1133: “People who say Christ will never return to earth are wrong”, 1233: “The claim that belief in God limits human knowledge is misguided”, 1073: “That God only exists in people's imaginations is doubtful”, and 1063: “The claim that Jesus was just an influential man in history, like many others, is untrue”).

**Table 4:** Internal consistency of the Cambridge Psycholinguistic Inventory of Christian Beliefs (Study 2)

	**Religious**					**Moral**	**Scientific**	**Everyday**	**Full inventory**
	Strong- Minded Atheists	Moderate Atheists	Moderate Christians	Strong- Minded Christians	All				
**Participants,** ***N***	10	10	10	10	40	40	40	40	40
**Inventory Items,** ***N***	100	100	100	100	100	100	100	100	400
**Items removed,** ***N***	0	0	0	0	0	2	2	14	18
**Cronbach’s alpha**	0.95	0.94	0.98	0.57	1	0.93	0.92	0.90	0.98

### Test–retest reliability of the Cambridge Psycholinguistic Inventory of Christian Beliefs

**Fig. 4:**
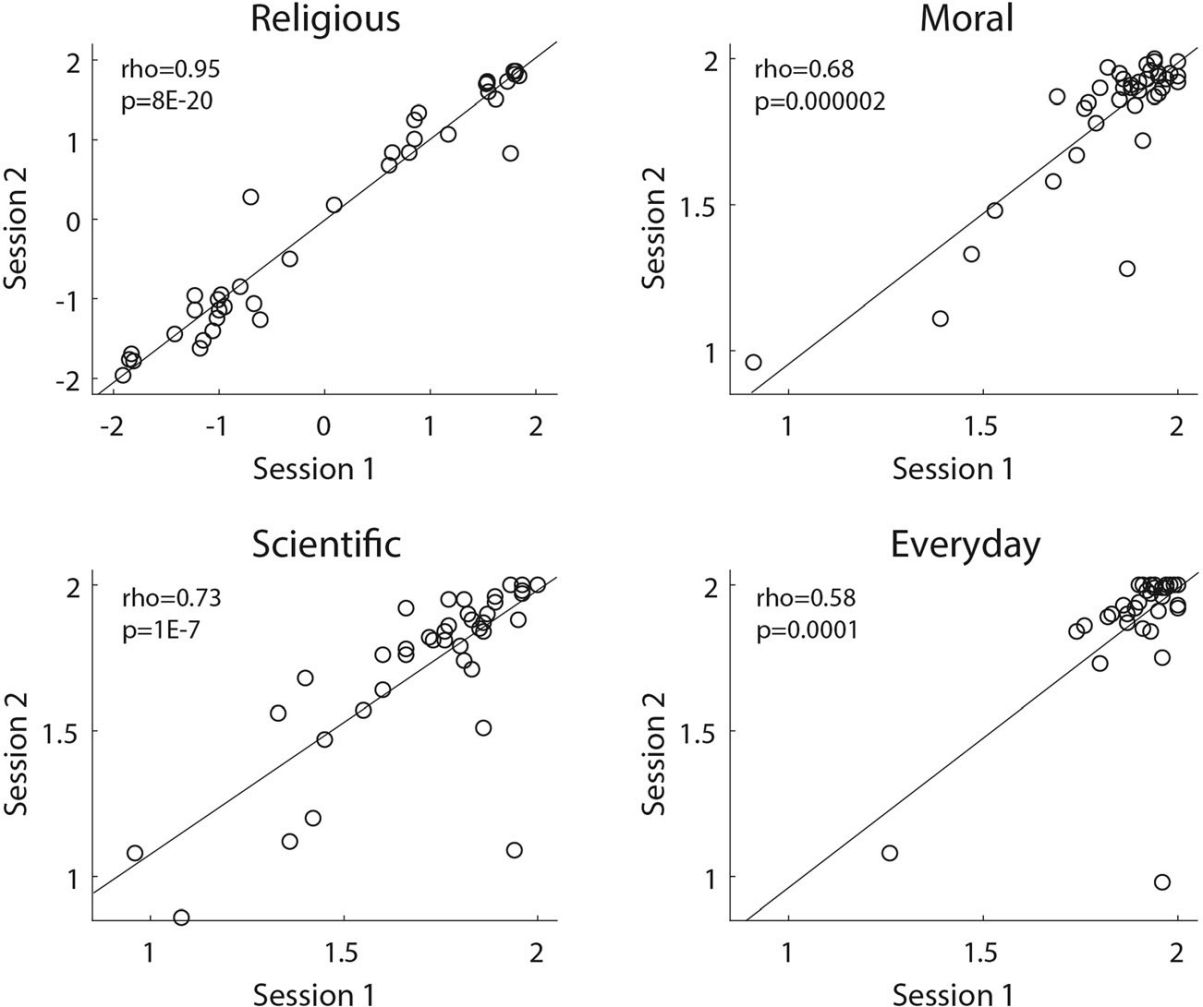
Association between the mean scores of the Cambridge Psycholinguistic Inventory of Christian Beliefs categories obtained on session 1 and session 2 (*N* = 39, Study 2). To calculate the mean score, responses to (Christian) Disagree statements were reversed. While test–retest reliability was assessed by calculating ICC, Spearman rank-order correlation coefficients are reported in this figure.

For one participant, most of the trials were registered as unresponsive during Session 2 (377 out of 400), most likely due to a keyboard malfunction. Hence, we excluded this participant from the test–retest reliability analysis, with a remaining total of 39 participants. When comparing the mean scores of the CPICB between Session 1 and Session 2, ICC values indicated excellent test–retest reliability for the Religious and Moral categories, good reliability for the Scientific category, and fair reliability for the Everyday category (see Table 5 and Fig. 4).

**Table 5:** Test–retest reliability of the Cambridge Psycholinguistic Inventory of Christian Beliefs

CPICB category	Intraclass correlation	95% Confidence interval		*F* test with true value 0		
		*Lower bound*	*Upper bound*	*F*	*df*	*p*
Religious	.974	.951	.986	74.860	38	2.7E−26
Moral	.843	.721	.914	11.696	38	4.7E−12
Scientific	.747	.566	.859	6.766	38	2.1E−8
Everyday	.524	.252	.719	3.161	38	0.0003

## Discussion

Here we present a new instrument—the *Cambridge Psycholinguistic Inventory of Christian Beliefs—*suitable for studying Christian religious beliefs in an experimental setting involving behavioural and neuroimaging paradigms. We developed and tested the CPICB over the course of two studies, ensuring that it meets the six criteria set out at the beginning of the project (see Introduction). In particular, we created a set of 100 audio-recorded religious (dis)belief statements, which can be experimentally contrasted with control sets of sentences reflecting moral beliefs (*N* = 100), abstract scientific knowledge (*N* = 100) or empirical facts about everyday life (*N* = 100). The critical word that reveals the meaning of each sentence is always placed at the end, thereby enabling temporally precise measurement of reaction times or neurophysiological responses. Each statement has two orthogonal versions constructed with identical strings of words, differing only in the final critical words so that one version is expected to elicit an Agree response, and the other a Disagree response. This way, the CPICB ensures that baseline periods of Agree and Disagree conditions are psychoacoustically and linguistically perfectly matched up to the moment of the presentation of critical words. Furthermore, each pair of statements has a “sister pair” of statements with reversed association between the two critical words and Agree/Disagree responses, which allowed us to match psychoacoustic and linguistic properties of critical words across Agree and Disagree conditions. Finally, the critical words are matched across religious, moral, scientific and everyday conditions for word frequency, length and sound intensity.

The CPICB shows robust psychometric properties, including its construct validity, internal consistency and test–retest reliability. Mean responses to the CPICB religious (dis)belief statements were very strongly associated with the mean scores of the Christian Orthodoxy Scale (Fullerton & Hunsberger, 1982), with correlation coefficients being > 0.9 in both Studies 1 and 2. We thus conclude that the CPICB is a valid tool to measure fundamental Christian beliefs. In future studies using the CPICB, participants can be recruited based on their responses either to the full Christian Orthodoxy Scale (Fullerton & Hunsberger, 1982) or its short version (Hunsberger, 1989). We also observed excellent test–retest reliability for the Religious statements of the CPICB, confirming that the religious belief statements were understandable and participants provided highly consistent responses on two separate occasions which transpired two or more weeks apart.

While there was excellent internal consistency for each category of the CPICB when testing a full group of participants (*N* = 20 in Study 1 and *N* = 40 in Study 2), we observed that internal consistency was not stable when tested within small subgroups (*N* = 10). In Study 1, Strong-Minded Christians (Cronbach’s alpha = 0.96) showed higher consistency than Strong-Minded Atheists (Cronbach’s alpha = 0.76), whereas in Study 2, Strong-Minded Atheists (Cronbach’s alpha = 0.95) showed higher consistency than Strong-Minded Christians (Cronbach’s alpha = 0.57). It seems that several Christian participants rated a small proportion of Religious items very unexpectedly compared to the rest of the Strong-Minded Christians. Given a large inter-individual variability and the subjective nature of religious beliefs, it is possible that a similar deviance from predicted responses and low internal consistency of Religious items is unavoidable when testing a small group of participants. Another factor which might explain the unexpected responses from the Christian participants is that the formulation and selection of religious statements were informed by a relatively small sample of theologians and Christian “experts”. Because there is a high number of distinct and varied Christian theological viewpoints, just as internal consistency is difficult to achieve within a small group of participants, so too is consistency difficult to achieve amongst a small group of experts. Finally, it is important to highlight that the estimate of Cronbach’s alpha lacks both power and precision when the sample size is very small (Bonett, 2002; Feldt & Ankenmann, 1999), which was likely the case when testing participant subgroups in the present study. It is also possible that we overestimated Cronbach’s alpha, and smaller reliability coefficients could be expected with a larger sample size (Javali et al., 2011). Nevertheless, we decided to carry out and include subgroup results because participant samples of a similar size are likely to be used in neuroimaging experiments using CPICB. For researchers that require high internal consistency within a small group of participants, e.g. *N* < 20, we recommend assessing internal consistency in their own dataset, removing problematic items one by one until satisfactory internal consistency is observed.

A major strength of the CPICB is that statements are carefully controlled and items are closely matched across conditions, with each item being presented in almost identical “agree” and “disagree” versions, and identical critical word lists used across agree and disagree versions (see details in Study 1, Methods). These measures make the CPICB suitable for neurocognitive studies where such matching is necessary in order to isolate the brain response to a specific difference between two sentences while also reducing the variation between conditions that might otherwise occur as a result of a participant having different levels of familiarity with the content or words of particular items. However, future researchers should be aware that while the sets of critical words have been matched across conditions for word frequency, intensity (dB) and length in syllables (see Table 2), other lexical differences remain which may need to be controlled for depending on the research question under investigation. For example, critical words are not matched for word category, concreteness (Holcomb et al., 1999; although of noun pairs used as critical words only two pairs out of 25 are abstract rather than concrete) or effects of valence (Jiang et al., 2014). Due to inherent differences between the four categories of items, the critical words used for Religious and Moral items tend to be more strongly valenced (e.g. wrong/right, correct/incorrect, anger/tolerance), while those used in Scientific and Everyday items are more neutral (e.g. hot/cold, sleepy/hungry, metal/paper).

The advantage of psychoacoustic and linguistic matching of long statements comes at the cost that it can be difficult to construct sets of sentences (such as the sister-pair sets shown in Table 1) that meet these technical requirements while sounding equally natural in each condition. This was particularly the case with Religious items where it was necessary to elicit the required opposing responses from Christians and Atheists. In creating the CPICB, we aimed to focus on and measure whether or not a Christian holds a particular religious belief, and characterised the Atheist viewpoint in terms of a lack of such Christian beliefs. Consequently, in order to create an item that an Atheist would disagree with which ends with a negative critical word (i.e. the third of the four sentences shown in Table 1), it was often necessary for the main part of the sentence to express a negative belief or action, such as *Ignoring the Bible as the most important source of life instructions is unwise*. This led to some awkward double-negative sentences which participants reported in piloting were difficult to process in the time available, for example *The belief that prayers are never answered by God is untrue*. Through repeated piloting and revision of materials, we improved these sentences. Where possible we replaced negative verbs with verbs such as “ignoring” or “rejecting” so that the first negative part of the sentence was, at least in grammatical terms, presented positively, and found other ways to express negative concepts such as replacing *That God doesn’t exist is doubtful* with *That God only exists in people's imaginations is doubtful.* While we were largely successful in replacing or rephrasing the most difficult-to-process sentences, it is still the case that some sentences of this type are slightly more complicated to process. Of the six items that showed lower internal consistency in Strong-Minded Christians, five of these were of this “Christian Agree/Atheist Disagree plus negative critical word” type, which suggests that the more complex sentence structure may have led several Strong-Minded Christians to misinterpret the meaning of the sentence under time constraints in Study 2. Arguably, it could be that hearing a seemingly anti-Christian main body of a sentence may cause Strong-Minded Christians distress, in which case they may overlook the meaning of the critical word and respond instead with their feelings about the main body of the sentence. It could also be that it is more onerous to revise a negative view than a positive view, so this coupled with the stress of a complex sentence and the stress of hearing a negation of your faith may contribute to misinterpretations. Importantly, a subgroup of Atheists rather than Christians showed lower internal consistency in Study 1. As all subgroups were matched by IQ, this is likely a random effect caused by some participants finding some sentences harder to process when facing the situational demands of the laboratory experiment.

Another area where the creation of perfectly matched sentences has had to be balanced with naturalness is in the splicing of the audio files. As files are spliced so that items in each condition are identical up to the critical word, a researcher can be confident that any response differences across conditions are due to the critical word alone, and not to any minor intonation/volume/emphasis differences in the main part of the sentence. However, due to the way that the pronunciation of a word in connected speech is influenced by the words preceding and following it, in some cases it required compromise in order to ensure that the baseline section had the same duration over all four statements in a sister-pair set. For example, due to catenation between a final consonant and an initial vowel in connected speech, the “is” in “is wise” is pronounced differently from the “is” in “is unwise”, as in the latter there is no gap between the two words. In such cases we digitally manipulated the baseline in order to find the best fit that allowed an identical baseline to be used in both versions of the sentence, while retaining the natural rhythm and sound of both. As our pre-experiment checks showed that naive participants were occasionally able to identify a splice junction, this raises the question of whether participants’ natural processing could be influenced by (possibly subconscious) awareness of these minor changes to the natural timing of the sentence. While this may be an issue in participants who have direct experience in splicing audio materials or are otherwise hyper-aware of connected speech patterns, the fact that 387/400 items were rated by all 4 raters as sounding completely natural satisfies us that this is unlikely to be an issue in naive participants. In addition, all participants in both study 1 and 2 were asked for their impressions at the end of the experiment, and none reported detecting anything anomalous with regard to the naturalness of the audio files.

While listening to a sentence, certain expectations naturally build up regarding how the sentence will end. Kutas and Hillyard (1984) showed that the N400 response to the final word in a sentence is strongly affected by its cloze probability, i.e. the probability of that particular word completing the sentence, with stronger N400 responses to words that were not predicted. For this reason, in future psychometric or psycholinguistic work using the CPICB, it would be informative to assess the predictability of critical words (as in Fondevila et al., 2016) in order to assess differences across sentence types (Religious, Moral, Scientific, Everyday) and agreement types (Agree vs Disagree sentences) with regard to the extent to which participants’ expectations of how the sentence will end are violated. Recent advances in computer modelling have led to the development of neural language models that predict the probability of a word based on the sentence context, and these predictability estimates have been shown to closely match with ERP responses (Helibron et al. 2019; Merkx & Frank, 2020). This opens up avenues for future research where word predictability could be statistically controlled for without the need for data collection from human participants. We would expect that predictability would be substantially lower in Disagree items than Agree items for Moral, Scientific and Everyday items, and that for Religious items it would depend on the Religious viewpoint of the participant (with items the participant agrees with again having a higher predictability).

One of the key limitations of the CPICB is its narrow focus on only one dimension of religiosity—explicit religious beliefs—among Christians. Relatively recent developments in the study of Christianity (or, as some scholars are careful to specify, the study of *Christianities*) have highlighted the intricate nuances and diversities that exist across the spectrum of Christian beliefs and practices (Cannell, 2006; Robbins, 2014). Christianity is indelibly shaped by the particular cultures in which it exists, and it would thus be extremely problematic to assume that all Christian individuals or all Christian communities embrace and adhere to the same precise doctrines. Furthermore, preoccupation with doctrinal belief is of particular importance to Protestant Christians (Harding, 2001; Luhrmann, 2012; Webster, 2013), whereas *belief* is not consistently interesting or significant for individuals from other religious contexts (Pouillon, 2016; Ritchie, 2002; Coleman, 2009). Indeed, even within some Protestant contexts, belief is not always a central indicator of one’s religious commitment or fervour (Howell, 2007; Ruel, 1982) and thus it cannot be assumed that one’s doctrinal beliefs are undeniable indicators of one’s religious commitments. It is important for us to highlight this insight that doctrinal belief is not always the central dimension of religiosity, precisely because our study was designed to study an individual’s inclination to either agree with or differ from a particular religious belief statement.

The sheer diversity of doctrinal beliefs within the various expressions of Christianity worldwide make it difficult, if not impossible, to speak of “Christian belief” as if it were a homogeneous and monolithic phenomenon. The particular theological viewpoints which informed our understanding of Christian beliefs were located within the cultural and theological milieus of Cambridge, UK, which is a progressive university town. There are a range of Christian theological viewpoints which were not explicitly foregrounded in our questions and it might very well be the case that an individual who strongly self-identifies as a Christian would not score as such upon using our inventory. One wonders, for example, if Christians whose theological and cultural contexts are significantly different from those of the Christians who guided our statements (see, for examples of strikingly different Christian theologies, the ethnographic accounts of Bauman (2015), Roberts (2016), Robbins (2014), and others who have studied Christian communities in non-Western cultural milieus) would score strongly as a “Christian” on the CPICB. We do not take this to indicate that the CPICB is inaccurate; rather, we take it as a reflection of the sheer diversity of Christianities that exist across the globe. In this vein, it is useful to bear in mind that the CPICB was developed with specific theologies as a point of reference for what it entails to be a Christian. It is noteworthy that, while the majority of our participants self-identified with Protestant forms of Christianity, we had participants who self-identified with other Christian denominations; the CPICB worked equally well amongst all participants. However, the significant rise of Pentecostal Christianity and Charismatic Christianity worldwide might not be well-suited to the CPICB questions, which are more angled towards a few particular types of Christian denominations in the UK. In other words, the definition of Christianity that we worked with was undergirded by two premises: firstly, there was an assumption that belief itself is a central way of determining Christian identity. Secondly, particular doctrinal beliefs were foregrounded over others.

With the above-mentioned caveats in mind, we nonetheless feel that it has been important and valuable to focus on individuals’ responses to statements of (religious) belief, and to construct those statements of belief with reference to the specific theological milieus that we did. Pragmatically, it will be easier to carry out quantitative group-level studies with participants committed to fundamental theologies, which allows us to predict participants’ answers and plan group comparisons. Contrary to this, participants recruited from communities with more liberal or less defined theologies would likely provide very diverse answers to the same questions, making it very hard to contrast groups of religious and non-religious individuals.

Finally, even though we observed an excellent internal consistency of responses to religious statements at a full group level in both studies, lower consistency within smaller subgroups suggests that the inventory may cover beliefs that yield varying responses within and across individuals. When developing the CPICB, we aimed to cover five broad categories of Christian beliefs, namely anthropological beliefs, God attributes, prophecies and eschatology, supernatural agents, and miracles (see Table S2). Given that these categories reflect different dimensions of Christian theology, individuals may be more likely to agree with some items, e.g. statements regarding God attributes, than with other items, e.g. statements about biblical miracles. For instance, when piloting the initial version of the inventory, we noted that statements regarding Satan and devils yielded very contradictory responses from Christian participants, and eventually we removed these items from the CPICB. It is however likely that the remaining items would form distinct clusters of relatively weaker or stronger beliefs, especially among less fundamental participants. This should be investigated in the future using factor analysis. We could not carry out factor analysis in the current experimental study due to the sample size. It is recommended that a ratio of the number of cases to the number of variables involved in the factor analysis would be 5–15 participants per variable (Hatcher, 1994; Nunnally, 1978; Pett et al., 2003). With 100 items in the Religious condition, we would need at least 500 participants. Given that it is not feasible to run lab experiments with such a large group of participants, a more suitable approach would be a follow-up online study asking a larger sample of participants to respond to 100 statements of religious beliefs. Identified dimensions of Christian beliefs may also have implications for neurophysiological research, e.g. distinct inventory components may be associated with different degrees of violation of the semantic predictions or affective responses.

We conclude that the CPICB is a reliable tool and we hope it will facilitate new research lines in the experimental psychology and cognitive neuroscience of religion. The CPICB is available (open access) on the project’s OSF site: https://osf.io/t85mr/ (DOI 10.17605/OSF.IO/T85MR). Users can freely download audio-recorded items of the CPICB in both their final version suitable for use in experiments, and in the earlier splicing version that allows researchers to extract and manipulate different parts of the sentences. In addition to the 400 items used in Study 2, we provide 80 items excluded after completion of Study 1 as well as an additional 16 items that can be used for practise at the beginning of experiments. Furthermore, we provide the full list of all items, information about temporal structure and duration of different parts of sentences, loudness and frequencies of critical words, and other meta information. Finally, we provide both raw and processed data of Studies 1 and 2.

## Supplementary Information

ESM 1 (DOCX 29 kb)

## Footnotes

1 The qualifier ‘Orthodoxy’ within the ‘Christian Orthodoxy Scale’ refers to the *orthodoxy* (that is, the authorized or widely accepted belief, theory and practice) of self-proclaimed Christians with reference to wider Christian theology. It should not be confused with Orthodox Christianity, which is a particular Christian tradition in the same way that Catholicism and Protestantism are particular traditions within Christianity. To avoid any confusion, in this paper we write ‘orthodox’ (lowercase) to refer to authorized and widely accepted Christian belief, and ‘Orthodox’ (uppercase) in the few cases where we refer to Orthodox Christians. In keeping with other literature, we retain the capitalization in ‘Christian Orthodoxy Scale’ when referring to the scale.

2 This question requires participants to indicate which of seven statements comes closest to expressing what they believe about God: 1.I don't believe in God. 2. I don't know whether there is a God, and I don't believe there is any way to find out. 3. I don't believe in a personal God, but I do believe in a Higher Power of some kind. 4. I find myself believing in God some of the time, but not at others. 5. While I have doubts, I feel that I do believe in God. 6. I know God really exists and I have no doubt about it. 7. Don't know.

3 In the Project registration, this information is not clearly presented, and it can be understood that positive/negative signs were reversed only for the Christian Disagree/Atheist Agree items in the Religious category. We confirm that positive/negative signs were also reversed for all Disagree items in the Moral, Scientific and Everyday categories.

4 Results of the Cognitive Reflection Test will be reported elsewhere.
