# Supplementary material for: Cambridge Psycholinguistic Inventory of Christian Beliefs: A registered report of construct validity, internal consistency and test–retest reliability
Source: Behav Res Methods. 2021 Jul 9;54(1):457–74. doi: 10.3758/s13428-021-01632-3 (PMC8863718; doi:10.3758/s13428-021-01632-3)
Supplement: Supplementary file 1 — (DOCX 29 kb) [file 13428_2021_1632_MOESM1_ESM.docx]

SUPPLEMENTARY MATERIALS

***Cambridge Psycholinguistic Inventory of Christian Beliefs*:**

**A registered report of construct validity, internal consistency and test–retest reliability**

Kaili Clackson^1,^*, Nadya Pohran^1,2^, Riccardo M. Galli^1,3^, Laura Labno^1,4^, Miguel Farias^5^,

Tristan A. Bekinschtein^1^, Valdas Noreika^1,6^*

^1^ Consciousness and Cognition Lab, Department of Psychology, University of Cambridge, Cambridge, United Kingdom

^2^ Faculty of Divinity, University of Cambridge, Cambridge, United Kingdom

^3^ Department of Experimental and Applied Psychology, Vrije Universiteit Amsterdam, Amsterdam, Netherlands

^4^ School of Psychology, Bangor University, Bangor, United Kingdom

^5^ Brain, Belief, and Behaviour Lab, Centre for Trust, Peace and Social Relations, Coventry University, Coventry, United Kingdom

^6^ Department of Biological and Experimental Psychology, School of Biological and Chemical Sciences, Queen Mary University of London, London, United Kingdom

* **Correspondence.** E-mail: kc496@cam.ac.uk (Kaili Clackson) and v.noreika@qmul.ac.uk (Valdas Noreika). Address: Department of Psychology, University of Cambridge, Downing Street, Cambridge CB2 3EB, United Kingdom

**Supplementary Tables**

**Table S1.** Exemplary sister-pair items of the Cambridge Psycholinguistic Inventory of Christian Beliefs

| **Category** | **Statement** | **Expected Response** |
| --- | --- | --- |
| *Religious* | Devoting time to building a good relationship with God is wise. | Christian Agree/ Atheist Disagree |
|  | Devoting time to building a good relationship with God is unwise. | Christian Disagree/ Atheist Agree |
|  | Ignoring the Bible as the most important source of life instructions is unwise. | Christian Agree/ Atheist Disagree |
|  | Ignoring the Bible as the most important source of life instructions is wise. | Christian Disagree/ Atheist Agree |
| *Moral* | Very rich people who avoid paying their taxes should be punished. | Agree |
|  | Very rich people who avoid paying their taxes should be praised. | Disagree |
|  | People who pick up plastic litter to recycle it should be praised. | Agree |
|  | People who pick up plastic litter to recycle it should be punished. | Disagree |
| *Scientific* | Compared to the size of planet Earth, Jupiter is larger. | Agree |
|  | Compared to the size of planet Earth, Jupiter is smaller. | Disagree |
|  | Compared to the size of the planet Jupiter, Earth is smaller. | Agree |
|  | Compared to the size of the planet Jupiter, Earth is larger. | Disagree |
| *Everyday* | If we want to borrow books we can go to the library. | Agree |
|  | If we want to borrow books we can go to the dentist. | Disagree |
|  | To avoid tooth decay we should regularly visit the dentist. | Agree |
|  | To avoid tooth decay we should regularly visit the library. | Disagree |

**Table S2.** Broad categories of items included in the Cambridge Psycholinguistic Inventory of Christian Beliefs

| **Religious** | **Moral** | **Scientific** | **Everyday** |
| --- | --- | --- | --- |
| - Anthropological beliefs - God attributes - Prophecies & eschatology - Supernatural agents - Miracles | - Behaviour to family and friends - Behaviour towards strangers - Positions of responsibility / Society - Behaviour towards the environment | - Astronomy - Physics - Earth Science - Biology - Medicine | - Uses of instruments - Cultural norms - Principles of the physical world (non-living) - Principles of the physical world (living) - Properties of familiar items |

**Table S3.** Selection requirements for pilot participants

| **Selection criteria** | **Atheist** | **Christian** |
| --- | --- | --- |
| *Self-identification* | Self-identify as Atheist | Self-identify as Christian |
| *Christian Orthodoxy Scale (Fullerton & Hunsberger, 1982)* | Christian Orthodoxy Scale* score between −72 and −45 | Christian Orthodoxy Scale* score between 45 and 72 |
| *Belief in God* | Selected response 1 or 2 from International Social Survey question about God  (i.e. “I don’t believe in God” or “I don’t know whether there is a God, and I don’t believe there is any way to find out”) | Selected response 5 or 6 from International Social Survey question about God  (i.e. “While I have doubts, I feel that I do believe in God” or “I know God really exists and I have no doubt about it”) |
| *Religious practices* | Selected response “never” or “once a year” from the question "How often do you take part in Christian practices (e.g., a personal prayer, a Bible reading/listening, or a church attendance)?" | Selected response “everyday” or “once a week” from the question "How often do you take part in Christian practices (e.g., a personal prayer, a Bible reading/listening, or a church attendance)?" |

**Note:** * Standard scoring for this scale yields a score between 24 (strongly Atheist) and 168 (strongly Christian). To make interpretation of scores more intuitive, we rescaled scores by subtracting 96, so that the most strongly Christian score was 72, and the most strongly Atheist score was −72, with 0 indicating a score in neither direction.

**Table S4.** Results of *t*-tests comparing linguistic and acoustic properties of critical words and sentences between conditions: Replaced Everyday sentences

|  | **Religious vs Everyday** | **Moral vs Everyday** | **Scientific vs Everyday** |
| --- | --- | --- | --- |
| **Critical word frequency** | *t*(167.49) = −.01  *p* = .99 | *t*(169.84) = .25  *p* = .80 | *t*(198) = −.94  *p* = .35 |
| **Critical word intensity (dB)** | *t*(198) = 1.05  *p* = .30 | *t*(198) = −.15  *p* = .88 | *t*(198) = 1.05  *p* = .30 |
| **Critical word length (syllables)** | *t*(198) = .55  *p* = .58 | *t*(198) = −.76  *p* = .45 | *t*(198) = −.19  *p* = .85 |
| **Critical word length (ms)** | *t*(198) = 1.54  *p* = .13 | *t*(198) = .84  *p* = .40 | *t*(198) = .20  *p* = .84 |
| **Length of sentences (words)** | *t*(198) = .28  *p* = .78 | *t*(193.05) = .68  *p* = .50 | *t*(198) = 1.12  *p* = .26 |

**Table S5.** Missing responses to the Cambridge Psycholinguistic Inventory of Christian Beliefs

|  | **Religious: Christian responses (*N* = 10)** | **Religious: Atheist responses (*N* = 10)** | **Moral:**  **Full group responses (*N* = 20)** | **Scientific: Full group responses (*N* = 20)** | **Everyday: Full group responses (*N* = 20)** |
| --- | --- | --- | --- | --- | --- |
| **Missing responses, *N*** | 11/1000 | 5/1000 | 11/2000 | 7/2000 | 8/2000 |
| **Non-responding participants, *N*** | 6/10 | 2/10 | 6/20 | 3/20 | 4/20 |
| **Misses per non-responding participant, *N*** | 1–3/100 | 2–3/100 | 1–3/100 | 1–4/100 | 1–4/100 |
| **Items missed by > 1 participant, *N*** | 0/100 | 0/100 | 1/100* | 0/100 | 0/100 |

**Note.** * Item was missed by 2 out of 20 participants.

**Table S6.** Participant information and test-retest interval in the four groups of participants

|  | **Strong-**  **Minded Atheists (*N* = 10)** | **Moderate**  **Atheists**  **(*N* = 10)** | **Moderate**  **Christians**  **(*N* = 10)** | **Strong-**  **Minded Christians**  **(*N* = 10)** | **All participants**  **(*N* = 40)** |
| --- | --- | --- | --- | --- | --- |
| Age (years), mean (*SD*) | 29.2 (8.6) | 25.9 (5.3) | 27.3 (7.1) | 28.7 (6.7) | 27.8 (6.9) |
| Male/Female, *N* | 2/8 | 3/7 | 3/7 | 4/6 | 12/28 |
| COS Score, mean (*SD*) | −64.4 (6.1) | −27.5 (13.3) | 25.8 (13.7) | 65.3 (7.2) | −0.2 (51.3) |
| Verbal IQ, mean (*SD*) | 124.8 (14.9) | 132 (19.9) | 126.4 (19.5) | 130.6 (16.3) | 128.5 (17.3) |
| Test–retest interval (days), mean (*SD*) | 15.4 (2.1) | 19.6 (6.4) | 19.4 (11.4) | 19.6 (5.6) | 18.5 (7.1) |

**Table S7.** Missing responses to the Cambridge Psycholinguistic Inventory of Christian Beliefs (*N* = 40, Study 2)

|  | **Religious: Strong-**  **Minded**  **Atheists (*N* = 10)** | **Religious: Moderate Atheists (*N* = 10)** | **Religious: Moderate Christians (*N* = 10)** | **Religious: Strong-**  **Minded Christians (*N* = 10)** | **Religious:**  **Full group (*N* = 40)** | **Moral:**  **Full group (*N* = 40)** | **Scientific: Full group (*N* = 40)** | **Everyday: Full group (*N* = 40)** |
| --- | --- | --- | --- | --- | --- | --- | --- | --- |
| **Missing responses, *N*** | 16/1000 | 9/1000 | 15/1000 | 4/1000 | 44/4000 | 10/4000 | 21/4000 | 7/4000 |
| **Non-responding participants, *N*** | 4/10 | 5/10 | 5/10 | 1/10 | 15/40 | 8/40 | 11/40 | 4/40 |
| **Misses per non-responding participant, *N*** | 1–9/100 | 1–3/100 | 1–8/100 | 4/100 | 1–9/100 | 1–2/100 | 1–6/100 | 1–3/100 |
| **Items missed by > 1 participant, *N*/100** | 3/100 | 1/100 | 1/100 | 0/100 | 11/100 | 1/100 | 3/100 | 0/100 |
